# Association among *VKORC1* rs9923231, *CYP4F2* rs2108622, *GGCX* rs11676382 polymorphisms and acute ischemic stroke

**DOI:** 10.1097/MD.0000000000034836

**Published:** 2023-08-25

**Authors:** Silvina Iluţ, Ştefan Cristian Vesa, Vitalie Văcăraş, Diana Şipoş-Lascu, Cristina Bârsan, Raluca Maria Pop, Sorin Crişan, Antonia Eugenia Macarie, Camelia Alexandra Coadă, Lăcrămioara Perju-Dumbravă, Dafin Fior Muresanu, Anca Dana Buzoianu

**Affiliations:** a Department of Neurosciences, “Iuliu Haţieganu” University of Medicine and Pharmacy, Cluj-Napoca, Romania; b Department of Pharmacology, Toxicology and Clinical Pharmacology, “Iuliu Haţieganu” University of Medicine and Pharmacy, Cluj-Napoca, Romania; c Department of Internal Medicine, “Iuliu Haţieganu” University of Medicine and Pharmacy, Cluj-Napoca, Romania; d Department of Geriatrics-Gerontology, “Iuliu Haţieganu” University of Medicine and Pharmacy, Cluj-Napoca, Romania; e Department of Medical and Surgical Sciences (DIMEC), University of Bologna, Bologna, Italy.

**Keywords:** *CYP4F2*, GGCX, ischemic stroke, rs11676382, rs2108622, rs9923231, *VKORC1*

## Abstract

Acute ischemic stroke is a major cause of morbidity and mortality worldwide, and genetic factors play a role in the risk of stroke. Single nucleotide polymorphisms (SNPs) in the *VKORC1, CYP4F2*, and *GGCX* genes have been linked to clinical outcomes, such as bleeding and cardiovascular diseases. This study aimed to investigate the association between specific polymorphisms in these genes and the risk of developing the first episode of acute ischemic stroke in patients without a known embolic source. This retrospective, cross-sectional, observational, analytical, case-control study included adult patients diagnosed with acute ischemic stroke. The SNPs in *VKORC1* rs9923231, *CYP4F2* rs2108622, *GGCX* rs11676382 genes were genotyped and analyzed together with the demographic and clinical factors of the 2 groups of patients. The presence of SNPs in *VKORC1* or *CYP4F2* genes significantly increased the risk of ischemic stroke in the context of smoking, arterial hypertension, and carotid plaque burden. The multivariate logistic model revealed that smoking (odds ratio [OR] = 3.920; *P* < .001), the presence of carotid plaques (OR = 2.661; *P* < .001) and low-density lipoprotein cholesterol values >77 mg/dL (OR = 2.574; *P* < .001) were independently associated with stroke. Polymorphisms in the *VKORC1* and *CYP4F2* genes may increase the risk of ischemic stroke in patients without a determined embolic source. Smoking, the presence of carotid plaques, and high low-density lipoprotein cholesterol levels were reconfirmed as important factors associated with ischemic stroke.

## 1. Introduction

Acute ischemic stroke is the leading cause of morbidity and mortality worldwide, accounting for approximately 80% of all stroke cases. Although several established risk factors for ischemic stroke, such as arterial hypertension (AH), smoking, and diabetes mellitus (DM) have been identified, the role of genetic factors in stroke risk remains an area of active research.^[1,2]^

Despite the significant progress made in the past 25 years to reduce acute strokes and preserve neurological function in affected patients, the most significant contribution one can make is prevention.^[3]^ Stroke has dropped from the third to the fifth leading cause of death in the United States over the past few decades; however, the aging population has increased the lifetime risk of stroke, making preventive care even more critical. Successful prevention depends on the appropriate application of various therapies, including antiplatelet and anticoagulant drugs, surgical interventions when necessary, and control of treatable risk factors (such as genetic risk factors).^[4]^ In this context, increasing attention has been paid to the role of single nucleotide polymorphisms (SNPs) in the pathophysiology and clinical relevance of various diseases, including acute ischemic stroke.^[5,6]^

The *VKORC1, CYP4F2*, and *GGCX* genes are 3 closely related genes involved in the metabolism of vitamin K, which plays a key role in blood coagulation and bone metabolism.^[7,8]^ *VKORC1* encodes vitamin K epoxide reductase, the target of oral anticoagulant therapy such as warfarin,^[9]^ whereas *CYP4F2* encodes an enzyme responsible for inactivating vitamin K1 to hydroxyvitamin K1, a process part of the vitamin K catabolism.^[10]^ *GGCX* encodes the gamma-glutamyl carboxylase enzyme, which is required for the posttranslational modification of vitamin K-dependent proteins, including blood coagulation factors and bone matrix proteins.^[11]^ Variants of these genes have been associated with a range of clinical outcomes, including bleeding disorders, bone metabolism disorders, and cardiovascular diseases. In particular, SNPs in the *VKORC1* gene have been shown to affect the efficacy and safety of warfarin therapy,^[9]^ while variants of *CYP4F2* and *GGCX* genes have been implicated in the pathogenesis of hypertension, thrombosis, and osteoporosis.^[11–14]^ Owing to the critical role of the key enzymes encoded by these genes, SNPs impacting their function lead to alterations in the metabolic pathways of vitamin K, which may become symptomatic in certain contexts, like in the case of the administration of various drugs such as oral anticoagulants. Numerous studies have shown the need to adjust drug doses in patients harboring such variants.^[15–17]^

Therefore, this study aimed to investigate a possible link between specific polymorphisms in the *VKORC1, CYP4F2*, and *GGCX* genes and the risk of developing a first episode of acute ischemic stroke in patients without a known embolic source. By identifying potential genetic risk factors associated with ischemic stroke, this study aimed to contribute to a better understanding of the underlying mechanisms of these events and contribute to the development of prevention strategies for at-risk individuals.

## 2. Material and Methods

### 2.1. Study design and patients

This was a retrospective, cross-sectional, observational, analytical, case-control study.

The study included adult patients diagnosed with acute ischemic stroke. This study was performed in accordance with the Declaration of Helsinki and was approved by the Ethics Committee of “Iuliu Haţieganu” University of Medicine and Pharmacy, Cluj-Napoca, Romania (no. 63/11.03.2019). The patients signed an informed consent form prior to inclusion in the study.

Acute stroke patients admitted at the Department of Neurology of the Emergency County Hospital in Cluj-Napoca between February 2019 and February 2020 were included in the study. The diagnosis of ischemic stroke was established by clinical and imaging criteria in accordance with the guidelines in place at that time.^[18]^

The control group included patients without acute ischemic stroke admitted to the Department of Internal Medicine, Cardiology, and Geriatric-Gerontology of the Municipal Clinical Hospital in Cluj-Napoca.

The study did not include patients with a history of ischemic stroke, transient ischemic attack, carotid stenosis >50%, hemorrhagic stroke, atrial fibrillation, cancer, autoimmune diseases, liver cirrhosis, or long-term anticoagulant treatment with vitamin K antagonists.

The following data were recorded for each patient: age, gender, living environment, smoking status, obesity (body mass index >30 kg/m^2^), presence of ischemic heart disease, history of myocardial infarction (MI), AH, heart failure, DM or dyslipidemia. Blood lipid profiles were also recorded. Carotid plaques were also observed. Ultrasound examination was performed at the Emergency County Hospital using a Samsung RS80 unit with a linear transducer with a frequency of 7 to 10 MHz. The ultrasound exam at the Municipal Clinical Hospital was performed using a Samsung RS85 unit with a linear transducer with a frequency of 7 to 10 MHz. The presence of carotid/femoral plaques was noted according to the following criteria: localized protrusion of the carotid wall, which was thicker than 1.5 mm, or more than 50% of the intima-media thickness of the adjacent area.^[19]^

### 2.2. DNA extraction and genotyping procedures

Peripheral blood was collected in a vacutainer containing ethylenediaminetetraacetic acid. DNA extraction was performed using a genomic DNA purification kit (Wizard Genomic DNA Purification Kit; Promega, Madison, WI) following the manufacturer instructions. Genotyping polymorphisms in the *VKORC1* rs9923231 (−1639G > A; VKORC1*2), *CYP4F2* rs2108622 (1347C > T), and *GGCX* rs11676382 (12970C > G) genes was performed as previously described.^[15,20]^

### 2.3. Statistical analysis

Statistical analysis was performed using MedCalc Statistical Software version 20.218 (MedCalc Software Ltd., Ostend, Belgium; https://www.medcalc.org; 2023). Qualitative data were presented as absolute and relative values. Normality of the distribution for quantitative data was assessed using the Shapiro–Wilk test and histogram visualization. Non-normal distribution was represented as median (the 25th and the 75th percentile). Comparisons between groups were performed using the Mann-Whitney or the chi-square tests, as appropriate. ROC analysis was used to establish a cutoff value for the association of low-density lipoprotein cholesterol (LDL-C) with stroke events. Variables that presented a *P* value of <.1 in the univariate analysis were used for the multivariate logistic regression. Multivariate logistic regression was used to identify variables that were independently associated with stroke. Statistical significance was considered at a *P* value of <.05 for univariate analysis, and <.006 for multivariate analysis (Bonferroni correction).

## 3. Results

The characteristics of the patients and controls included in this study are shown in Table 1. Both groups were comparable with respect to demographic profile. In the stroke group, there were significantly more smokers and patients with AH and a history of MI or ischemic heart disease, exceeding the threshold for statistical significance.

**Table 1:** Comparison between study groups.

Variables		Stroke group (N = 249)	Control group (N = 250)	*P* value
Age (median, IQR)		68 (56.5; 79)	67 (56; 75.2)	.2
Gender (N, %)	Male	107 (43)	116 (46.4)	.4
	Female	142 (57)	134 (53.6)	
Environment (N, %)	Urban	160 (64.5)	155 (62)	.6
	Rural	88 (35.5)	95 (38)	
Smoking (N, %)		78 (31.3)	31 (12.4)	<.001
Obesity (N, %)		120 (48.2)	113 (45.2)	.5
BMI (N, %)		26.8 (24; 32)	26.6 (24.3; 29)	.5
AH (N, %)		175 (70.3)	147 (58.8)	.01
HF (N, %)		21 (8.4)	16 (6.4)	.4
History of MI (N, %)		20 (8)	6 (2.4)	.009
IHD (N, %)		44 (17.7)	24 (9.6)	.01
DM (N, %)		49 (19.7)	34 (13.6)	.08
TC (mg/dL) (median, IQR)		193 (167; 218.7)	186 (152; 213)	.03
HDL-C (mg/dL) (median, IQR)		45 (36; 56)	45 (37.7; 54.2)	.5
LDL-C (mg/dL) (median, IQR)		119 (94.1; 142.4)	109.8 (79.4; 132.9)	.007
LDL-C > 77 mg/dL (N, %)		213 (85.2)	180 (72.3)	.001
TG (mg/dL) (median, IQR)		121 (85.5; 162)	116 (84; 156.5)	.7
Dyslipidemia (N, %)		158 (65.5)	149 (59.8)	.4
Carotid plaque (N, %)		142 (57)	92 (36.8)	<.001
*CYP4F2* (1347C > T) SNP (N, %)	w/w	107 (43)	126 (50.4)	.1
	w/m	112 (45)	104 (41.6)	
	m/m	30 (12)	20 (8)	
*CYP4F2* (1347C > T) SNP (N, %)	w/w	107 (43)	126 (50.4)	.1
	w/m or m/m	142 (57)	124 (49.6)	
*GGCX* (12970C > G) SNP (N, %)	w/w	219 (88)	205 (83.7)	.2
	w/m	30 (12)	40 (16.3)	
*VKORC1* (−1639G > A) SNP (N, %)	w/w	66 (26.5)	91 (36.4)	.04
	w/m	143 (57.4)	129 (51.6)	
	m/m	40 (16.1)	30 (12)	
*VKORC1* (−1639G > A) SNP (N, %)	w/w	66 (26.5)	91 (36.4)	.02
	w/m or m/m	183 (73.5)	159 (63.6)	

AH = arterial hypertension, BMI = body mass index, DM = diabetes mellitus, HDL-C = high-density lipoprotein cholesterol, HF = heart failure, IHD = ischemic heart disease, IQR = interquartile range, LDL-C = low-density lipoprotein cholesterol, m = mutated, MI = myocardial infarction, N = number, SNP = single nucleotide polymorphism, TC = total cholesterol, TG = triglycerides, w = wild-type.

The *VKORC1* −1639G > A mutant and *CYP4F2* 1347C > T m/m genotypes were more frequently encountered in the stroke group, although in the case of *CYP4F2* m/m, the threshold for statistical significance was slightly exceeded. For LDL-C, a cutoff value of 77 mg/dL was calculated for association with stroke (AUC 0.573; *P* = .007). Non-calcified plaques were present in 34 (23.9%) patients in the stroke group and in 15 (16.3%) patients in the control group (*P* = .03).

We calculated the OR for the variables that were significantly more frequent in the stroke group: smoking (3.222 (95%CI 2.031; 5.113), *P* < .001), AH (1.657 (95%CI 1.144; 2.400), *P* = .008), history of MI (3.552 (95%CI 1.401; 9.001), *P* = .008), LDL-C > 77 mg/dL (2.207 (95%CI 1.413; 3.447), *P* = .001), carotid plaque (2.279 (95%CI 1.591; 3.264), *P* < .001), *VKORC1* (−1639G > A) polymorphism w/m or m/m genotype (1.587 (95%CI 1.084; 2.324), *P* = .01).

We assessed the possible interaction between mutations in the *VKORC1* and *CYP4F2* genes and the variables that were statistically significant in the univariate analysis (Table 2). *VKORC1* and *CYP4F2* mutational status was dichotomized as either no mutation or monoallelic or biallelic mutations. For almost all variables, the presence of *VKORC1* or *CYP4F2* mutations significantly increased the risk of ischemic stroke.

**Table 2:** Interaction assessment between VKORC1 and CYP4F2 and variables of interest.

Variable 1	Variable 2	OR (95%CI)	*P* value
Smoking	*CYP4F2*	3.913 (2.050; 7.468)	<.001
Smoking	*VKORC1*	2.802 (1.645; 4.773)	<.001
Smoking	*CYP4F2* or *VKORC1*	3.114 (1.886; 5.141)	<.001
History of MI	*CYP4F2*	4.116 (0.865; 19.580)	.07
History of MI	*VKORC1*	2.842 (0.893; 9.051)	.07
History of MI	*CYP4F2* or *VKORC1*	2.919 (1.035; 8.232)	.043
AH	*CYP4F2*	1.731 (1.189; 2.521)	.004
AH	*VKORC1*	1.781 (1.247; 2.543)	.001
AH	*CYP4F2* or *VKORC1*	1.753 (1.228; 2.502)	.002
LDL-C > 77 mg/dL	*CYP4F2*	1.334 (0.935–1.903)	.1
LDL-C > 77 mg/dL	*VKORC1*	1.093 (0.769–1.554)	.6
LDL-C > 77 mg/dL	*CYP4F2* or *VKORC1*	1.162 (0.801; 1.688)	.4
Carotid plaque	*CYP4F2*	2.176 (1.428; 3.314)	<.001
Carotid plaque	*VKORC1*	2.427 (1.661; 3.546)	<.001
Carotid plaque	*CYP4F2* or *VKORC1*	2.468 (1.710; 3.564)	<.001

AH = arterial hypertension, CI = confidence intervals, LDL-C = low-density lipoprotein cholesterol, MI = myocardial infarction.

The multivariate logistic model, as shown in Table 3, revealed that smoking, the presence of carotid plaques, and LDL-cholesterol values >77 mg/dL were independently associated with stroke. Polymorphisms in the *VKORC1* (−1639G > A) m/m genotype, the *CYP4F2* (1347C > T) w/m and m/m genotypes, and history of MI were also linked to stroke, but following Bonferroni correction, the *P* value threshold was slightly exceeded.

**Table 3:** Multivariate logistic regression for ischemic stroke.

Variables	B	*P* value	OR	95% CI OR	
				Min	Max
Smoking	1.366	<.001	3.920	2.385	6.444
AH	0.207	.3	1.230	0.801	1.888
History of MI	1.145	.02	3.141	1.152	8.567
DM	0.244	.3	1.276	0.749	2.174
Carotid plaque	0.979	<.001	2.661	1.764	4.014
*CYP4F2* (1347 C > T) SNP w/w		.04			
*CYP4F2* (1347 C > T) SNP w/m	0.434	.04	1.543	1.020	2.332
*CYP4F2* (1347 C > T) SNP m/m	0.671	.051	1.957	0.997	3.841
*VKORC1* (−1639G > A) SNP w/w		.07			
*VKORC1* (−1639G > A) SNP w/m	0.320	.1	1.376	0.888	2.133
*VKORC1* (−1639G > A) SNP m/m	0.700	.02	2.014	1.088	3.728
LDL > 77 mg/dL	0.945	<.001	2.574	1.583	4.184
Constant	1.444	<.001	4.237		

AH = arterial hypertension, B = regression coefficient, CI = confidence intervals, DM = diabetes mellitus, m = mutated, MI = myocardial infarction, OR = odds ratio, SNP = single nucleotide polymorphism, w = wild-type.

## 4. Discussion

Our study showed that smoking, presence of carotid plaques, and high LDL-C levels were significantly more frequent in patients with ischemic stroke without a determined embolic source. The *VKORC1* and *CYP4F2* homozygous genotypes were also more frequent in stroke patients and increased the risk of stroke when they were found in patients with specific comorbidities or conditions. However, in the case of multivariate analysis, their effect was not statistically significant following the Bonferroni correction. This is the first study on patients with ischemic stroke that has examined the role of polymorphisms in the *VKORC1, CYP4F2*, and *GGCX* genes together. Previous studies have only considered the *VKORC1* gene and the *CYP4F2* gene separately.

Smoking was the most important variable associated with acute ischemic stroke in our study, as it was almost 4 times more frequent in the stroke group than in the control group. Data from 2019 showed that at least 1 billion people were active smokers, and more than 8 million people died every year from smoking-related causes.^[21]^ Several studies have demonstrated that smoking is a strong risk factor for ischemic stroke, as current smokers have 2 to 6 more chances of developing stroke.^[22–26]^ Smoking leads to accelerated atherosclerosis, including ATS of the carotid and cerebral arteries, with high levels of oxidized LDL-C, homocysteine, and other proinflammatory markers. Changes in the arterial walls lead to endothelial dysfunction, which translates into reduced vasodilation through impaired nitric oxide production and increased rigidity. Arterial plaques that appear in the carotid and brain arteries can cause several problems, including decreased brain perfusion to thrombosis or impaired endogenous fibrinolysis.^[27–31]^

In our study, carotid plaques were independently associated with ischemic stroke, as they were present in more than 50% of the cases. Carotid plaques account for approximately 15% of ischemic strokes. The carotid bifurcation is the most common location of carotid plaques.^[32]^ Numerous studies and metanalysis established the increased risk of stroke or transient ischemic attacks in patients with carotid plaques, especially in high-risk plaques.^[33–35]^ Non-stenotic carotid plaques were present in approximately 40% of patients with embolic stroke of an undetermined source, similar to our study.^[36]^ The mechanisms by which carotid plaques increase the risk of stroke are complex, and most of them have to do with the changes in vessel walls due to atherosclerosis, which lead to an unstable plaque. There is a complex interaction between inflammation, degradation of extracellular matrix components, neovascularization of the plaque, and the presence of comorbidities such as diabetes or chronic kidney disease.^[37]^ Most of the plaques from our stroke patients were calcified, but this is partially explained by the increased frequency of homozygous and heterozygous genotypes of *VKORC1* and *CYP4F2* polymorphisms. We already proved that *CYP4F2* and *VKORC1* polymorphisms are associated with an increased risk of carotid plaques.^[38]^ There are studies showing that calcified plaques also have higher probability of rupture.^[39–41]^

High LDL-C levels were more frequently found in patients with stroke. Considering that our patients were at their first episode of stroke, without an apparent embolic source, an increased risk for those with an LDL-C level >77 mg/dL seems to be in accordance with the literature. The relationship between dyslipidemia and stroke risk has been well-established. Many studies have shown that intensive lipid-lowering therapy with a high dose of statins or a combination of statins with ezetimibe/proprotein convertase subtilisin/kexin type 9 (PCSK9) inhibitors prevents recurrent stroke after transient ischemic attack and ischemic stroke. The recommended target for LDL-C in people with very high cardiovascular risk is <55 mg/dL. A drop below 70 mg/dL protected the patients by lowering the risk of subsequent cardiovascular events, although some studies showed an increased risk of hemorrhagic stroke.^[42–44]^

The *VKORC1* and *CYP4F2* homozygous genotypes were associated with an increased risk of stroke, even though the threshold for statistical significance was slightly exceeded in the multivariate model. When we considered their combined effect with various variables (smoking, history of MI, carotid plaques, etc), they increased the probability of stroke. The *VKORC1* − 1639G > A polymorphism did not increase the risk of ischemic stroke in 1 study. However, the study had a smaller number of patients and included patients with atrial fibrillation, which can diminish the impact of the mutation.^[45]^ Other studies have shown that *VKORC1* variants are associated with stroke recurrence and thrombosis. The studies included only patients treated with vitamin K antagonists, which are associated with vessel calcification and might constitute a bias.^[46,47]^ The association between *VKORC1* G-1639A polymorphism and ischemic stroke was reported in Ukrainian patients without atrial fibrillation or vitamin K antagonists treatment.^[48]^ Because *VKORC1* is involved in the vitamin K cycle, mutations in this gene result in lower levels of vitamin K-dependent proteins, including those implicated in the inhibition of vascular calcification.^[49]^ A low level of vitamin K is associated with proteoglycan loss, a part of the extracellular matrix that plays an important role in the regulation of vascular permeability.^[50,51]^ We previously hypothesized that *VKORC1* and *CYP4F2* polymorphisms interact with cardiovascular disease, enhance the proinflammatory status, and increase the formation of carotid plaques.^[37]^ The synergistic effect of these proposed mechanisms may result in carotid plaques being more prone to rupture.

Genetic variants of *CYP4F2* have been found to increase susceptibility to ischemic stroke in various populations.^[52–54]^ CYP4F2 may increase the risk of ischemic stroke through vasoconstriction, high oxidative stress and endothelial dysfunction.^[55,56]^

The limitations of our study include the small sample size due to extensive exclusion criteria and the retrospective nature of the study. This moderate number can be attributed to the COVID-19 pandemic, as we were forced to end our study in February 2020, much earlier than originally planned. We could not exclude patients with important risk factors for accelerated atherosclerosis such as smoking, AH, or DM.

## 5. Conclusions

The *VKORC1* rs9923231 and *CYP4F2* rs2108622 polymorphisms may increase the risk of ischemic stroke in patients without a determined embolic source. The *GGCX* rs11676382 polymorphism did not increase the risk of ischemic stroke. Smoking, the presence of carotid plaques, and high LDL-C levels were reconfirmed as important factors associated with ischemic stroke.

## Author contributions

**Conceptualization:** Silvina Iluţ, Stefan Cristian Vesa, Sorin Crişan, Anca Buzoianu.

**Data curation:** Stefan Cristian Vesa, Vitalie Văcăraş, Cristina Bârsan, Raluca Pop, Camelia Coadă.

**Formal analysis:** Stefan Cristian Vesa, Camelia Coadă.

**Funding acquisition:** Silvina Iluţ, Stefan Cristian Vesa, Anca Buzoianu.

**Investigation:** Silvina Iluţ, Vitalie Văcăraş, Diana Şipoş-Lascu, Cristina Bârsan, Raluca Pop, Sorin Crişan, Antonia Macarie.

**Methodology:** Silvina Iluţ, Stefan Cristian Vesa, Raluca Pop, Sorin Crişan, Lăcrămioara Perju-Dumbravă.

**Project administration:** Silvina Iluţ, Diana Şipoş-Lascu, Dafin Mureşanu.

**Resources:** Raluca Pop, Lăcrămioara Perju-Dumbravă.

**Supervision:** Silvina Iluţ, Antonia Macarie, Lăcrămioara Perju-Dumbravă, Dafin Mureşanu, Anca Buzoianu.

**Validation:** Vitalie Văcăraş.

**Writing – original draft:** Silvina Iluţ, Stefan Cristian Vesa, Camelia Coadă.

**Writing – review & editing:** Silvina Iluţ, Stefan Cristian Vesa, Camelia Coadă, Anca Buzoianu.
